# Role of self-organising myddosome oligomers in inflammatory signalling by Toll-like receptors

**DOI:** 10.1186/s12915-019-0637-5

**Published:** 2019-02-20

**Authors:** Nicholas J. Gay

**Affiliations:** Department of Biochemistry, University of Cambridge, Cambridge, CB2 1GA UK

## Abstract

A paper published in *BMC Biology* characterises biophysically oligomeric and filamentous structures formed spontaneously by the Toll-like receptor signalling adaptor MyD88. Naturally occurring mutants of MyD88 that cause immunodeficiency are unable to form these structures. By contrast a somatic mutant that promotes the survival of tumour cells forms oligomers much more readily than the wild-type protein. These findings suggest that assembly of oligomeric MyD88 is critical for the regulation of inflammatory signalling.

## Commentary

**Fig. 1.**
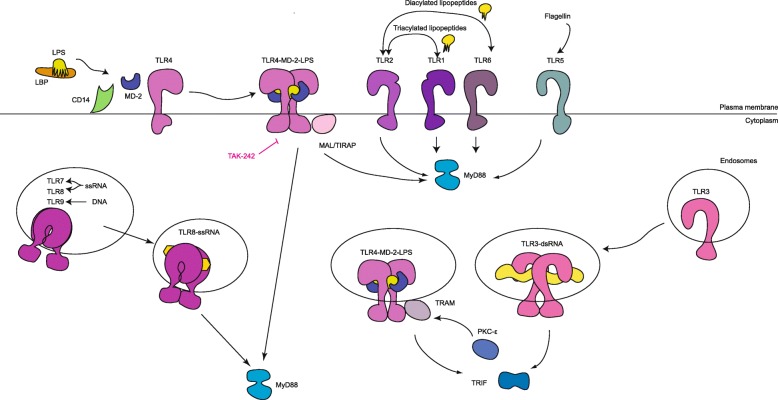
Overview of immune response signalling by Toll-like receptors. Toll-like receptors (TLRs) are present on the cell surface and in endosomes, where they detect microbial cell-wall components, non-self nucleic acids, or danger-associated self molecules. Upon stimulation, TLRs activate pathways that involve myeloid differentiation primary response protein 88 (MYD88) and/or TIR domain-containing adaptor protein inducing IFNβ (TRIF). MYD88 and TRIF nucleate signalling scaffolds, known respectively as myddosomes and triffosomes, that recruit kinases and activate downstream signalling pathways. Crosstalk with other signalling pathways ensures that the TLR signal is properly regulated and leads to apoptosis or cell survival, and the transcription of pro-inflammatory cytokines and chemokines, and type I interferons (IFNs). CD14, a coreceptor for LPS; LBP, LPS-binding protein; LPS, lipopolysaccharide; MAL/TIRAP, MYD88 adaptor-like protein; MD2, myeloid differentiation factor 2; PKCɛ, protein kinase Cɛ; TAK-242, TLR4 inhibitor; TRAM, TRIF-related adaptor molecule. Image and legend adapted from [1]

Innate immune and inflammatory signalling underlie a large burden of human disease ranging from sepsis to rheumatoid arthritis and neuronal degeneration. Thirty years ago Charles Janeway correctly predicted a central role in these signalling processes for what he termed pattern recognition receptors (PRRs), germline-encoded molecules that recognise conserved structures associated with microbial pathogens (PAMPS). One important group of PRRs is the Toll-like receptors (TLRs). These molecules respond, broadly speaking, to non-self lipids and nucleic acids presented at the cell surface or in endosomes, respectively (Fig. 1). Because of their central role in inflammatory signalling the molecular mechanisms of signal transduction by TLRs are of considerable interest and are therapeutic targets for novel anti-inflammatory medicines [1].

**Fig. 2.**
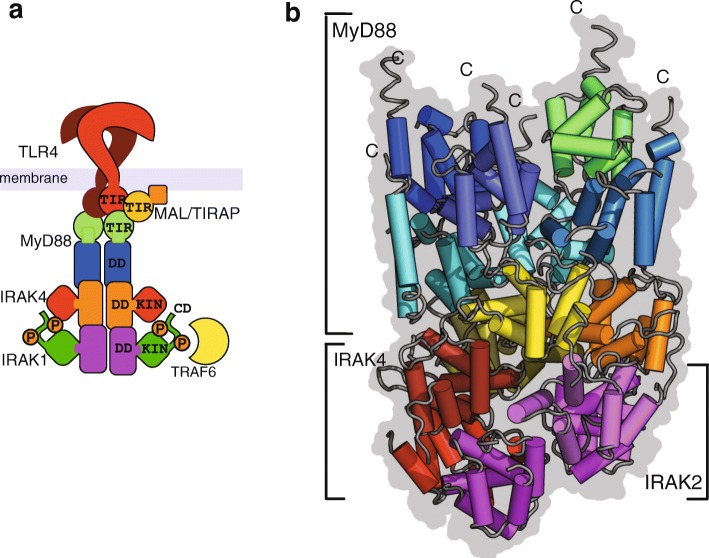
Death domain and TIR assemblies in Toll signalling through the myddosome. **a** An activated dimeric Toll-like receptor 4 (TLR4) showing the arrangement of its cytosolic TIR domains in association with Myd88 TIR domains, MyD88 death domains (DD), and interleukin-1 receptor-associated kinase (IRAK) kinase domains (KIN). **b** The myddosome DD assembly is shown with six MYD88 DDs (*blues* and *green*), four IRAK4 DDs (*red*, *orange* and *yellow*) and four IRAK2 DDs (*violet*). Image and legend adapted from [1]

PAMPs, such as lipopolysaccharide (LPS) from Gram-negative bacteria, bind directly to TLRs to induce formation of an active dimeric complex. Dimerization of the cytosolic TIR domains provides a transient nucleation signal for the assembly of an oligomeric scaffold, the myddosome, that is required for downstream signal transduction [2, 3]. The myddosome consists of the signal transducer MyD88 and IRAK kinases (Fig. 2). It has an unusual and variable stoichiometry of six to eight MyD88, four IRAK4 and four IRAK2 subunits [4]. A paper published in this issue of *BMC Biology* by Gambin and colleagues [5] provides new insights into how this complex may be assembled.

MyD88 consists of two modules, a death domain (DD) and a TIR domain. Dimerisation of the TLR TIR domains creates a new binding surface that can interact with the corresponding TIR domains of MyD88, nucleating the assembly of a closed myddosome complex. In resting cells it is thought that MyD88 is auto-inhibited, perhaps by interactions between the TIR and death domains. Transient interaction with the activated receptor is viewed as relieving this inhibition, allowing the formation of the helical myddosome assembly [6].

In the present study the authors have used an in vitro system to produce full-length MyD88 and in addition the isolated DD and TIR domain. This allows protein to be produced in a controlled manner and single molecule fluorescence methods were then used to assay the aggregation propensity of DDs, TIR domains and full-length MyD88. This analysis reveals that, at nanomolar concentrations, full-length MyD88 is able to form large polymers or filaments whereas only short oligomers of TIR domains are detected. At this low concentration the DD is also able to form oligomers, although these are much smaller than the polymers formed by the full-length protein.

Two mutations in MyD88 that cause immune deficiency in humans were then tested for their effect on the propensity of full-length MyD88 to oligomerise. Both of these, one in the DD and one in the TIR domain, abrogated polymer formation. A third variant, L252P in the TIR domain, is of particular interest because it is found as a dominant somatic mutation in many B-cell lymphomas [7]. The presence of L252P causes stimulus-independent activation of MyD88-directed signals and constitutive activation of NFκB, conferring a pro-survival phenotype in these tumours. Interestingly L252P MyD88 formed lower order oligomers compared to the wild-type but the formation of these short oligomers had a critical threshold of 2 nM, about 40-fold lower than that of the wild type. This result is consistent with previous studies indicating that the L252P MyD88 was able to form signalling myddosomes at endogenous concentrations in the absence of a nucleating signal from an activated TLR [8].

A key question unanswered by this study is the stoichiometry of endogenous MyD88 in unstimulated cells. Overexpression of MyD88 leads to the appearance of speckles in the cytosol, likely caused by oligomerisation, but endogenous MyD88 appears to be uniformly distributed. This might indicate that it forms only short oligomers rather than filaments or perhaps that it is monomeric. One published study provides evidence that MyD88 is monomeric or possibly dimeric in lysates prepared from resting cells, but this conclusion may be unreliable as preparation of the lysates could cause significant dilution of the MyD88 [9]. A more direct measurement of stoichiometry might be possible using single molecule imaging or super resolution microscopy.

The role played by oligomers and filaments in innate immune signal transduction has been an emerging theme over the last few years. These structures have been described as supra-molecular organising centres and are formed during activation of the TLR-TRIF and inflammasome NLRP3-ASC-Caspase1 pathways as well as in pro-inflammatory signalling through MyD88 [10]. A recent study solved the structure of a filament formed by another signalling adaptor protein that enhances signal transduction by TLR2 and TLR4, Mal/TIRAP [3]. Although the filaments produced by Mal/TIRAP in vitro are unlikely to represent a signalling scaffold in vivo, this study nevertheless identified TIR–TIR interactions that are probably critical for potentiating MyD88-driven signals. The study of Gambin and colleagues suggests that oligomers formed by other TLR signal transducers are also critical for signalling function. Structural analysis of the oligomers and filaments formed by both the full length MyD88 and the DD alone will provide further insights into the mechanism by which activated receptors nucleate TLR signalling as well as the role of auto-inhibition and polymerisation in this critical cellular process. It is also likely that small molecule inhibitors that target interfaces required for filament assembly can be developed as novel anti-inflammatory drugs.
